# Supplementary material for: Inherited polygenic effects on common hematological traits influence clonal selection on JAK2V617F and the development of myeloproliferative neoplasms
Source: Nat Genet. 2024 Jan 17;56(2):273–80. doi: 10.1038/s41588-023-01638-x (PMC10864174; doi:10.1038/s41588-023-01638-x)
Supplement: Supplementary file 1 — Supplementary Figs. 1–11 and Tables 1–15. [file 41588_2023_1638_MOESM1_ESM.pdf]

nature genetics

Article

https://doi.org/10.1038/s41588-023-01638-x

Inherited polygenic effects on common hematological traits influence clonal selection on *JAK2*^{V617F} and the development of myeloproliferative neoplasms

In the format provided by the authors and unedited

Table of Contents

Supplementary Figure 1 Flowchart of study design and data sets used in the study. 3
Supplementary Figure 2 Distribution of the VAF of *JAK2*-V617F G>T mutation in the genetically unrelated mutation carriers in the 200k UKBB-WES data (genetic relatedness <0.05; n = 540). 4
Supplementary Figure 3 Summary plot for *JAK2*-V617F mutation detection in the 200k UK Biobank WES data. 5
Supplementary Figure 4 Case-control GWAS of *JAK2*-V617F positivity in unrelated individuals in the UKBB-WES cohort (n = 1,125 cases vs. 338,919 controls). 6
Supplementary Figure 5 MR plots for the forward causality between a blood cell trait and *JAK2*-V617F positivity. 7
Supplementary Figure 6 MR plots for the reverse causality between *JAK2*-V617F positivity and a blood cell trait 8
Supplementary Figure 7 Ancestry of individuals in the MPN-patient cohort and the control set INTERVAL 9
Supplementary Figure 8 Polygenic germline determinants of normal blood cell traits and their contribution to MPN classification (ET versus PV) in the MPN-patient cohort (a; n = 581 ET, 112 PV and 30,305 controls) and the UKBB-MPN cohort (b; n = 156 ET, 161 PV and 161,872 controls). 10
Supplementary Figure 9 Time difference from blood sample collected to diagnosis in years for ET (n = 155) and PV (n = 161) patients in UKBB-MPN cohort. 11
Supplementary Figure 10 Proportions of PV and ET patients in the MPN-patient cohort (n = 581 for ET and 112 for PV) in each PGS quintile 12
Supplementary Figure 11 PGS distributions for the five identified haematological traits between cases in the UKBB-MPN cohort and controls in UKBB 13
Supplementary Table 1 Summary table of the UKBB-WES data across *JAK2*-V617F mutant reads groups. 14
Supplementary Table 2 Full names and abbreviations of 29 blood cell traits involved in the study. 15
Supplementary Table 3 Germline polygenic associations with *JAK2*-V617F positivity in the UKBB-*JAK2*-V617F cohort 16
Supplementary Table 4 Germline polygenic associations with *JAK2*-V617F positivity in the UKBB-*JAK2*-V617F cohort excluding MPN cases 17
Supplementary Table 5 Germline polygenic associations with *JAK2*-V617F positivity in the *JAK2*-V617F carriers in the full set of UKBB-WES. 18
Supplementary Table 6 Forward causal inference of *JAK2*-V617F positivity across four main MR methods based on the *JAK2*-V617F GWAS with 540 cases vs. 161,994 controls in UKBB-WES. 19
Supplementary Table 7 Forward causal inference of *JAK2*-V617F positivity across four main methods based on the *JAK2*-V617F GWAS in full UKBB (n = 1,125 cases vs. 338,919 controls). 20
Supplementary Table 8 Forward causal inference of *JAK2*-V617F positivity across four main methods with *JAK2*-V617F GWAS in full UKBB (n = 1,125 vs. 338,919) excluding instruments that are either associated with *JAK2*-V617F positivity at P <1e-6, in LD (LD r2 >0.01) with or within +/- 5Mb region of those variants. 21
Supplementary Table 9 Reverse causal inference of *JAK2*-V617F positivity and haematological traits across four main methods (#instruments = 11 with association P <1e-6; LD r2 <0.05) based on the *JAK2*-V617F-positivity GWAS in full UKBB (n = 1,125 cases vs. 338,919 controls). 22
Supplementary Table 10 Regression coefficients of significant (P < 0.0011 after Bonferroni correction) demographic, somatic mutation or germline variables for haematological traits in the MPN-patient cohort (corresponding to Figure 2c). 23
Supplementary Table 11 Regression coefficients of *JAK2* and PGS (P < 0.0063 after Bonferroni correction) in the UKBB-MPN cohort 24
Supplementary Table 12 Polygenic germline effects of normal blood cell traits on MPN in our MPN-patient cohort 25
Supplementary Table 13 Polygenic germline effects of normal blood cell traits on MPN in the UKBB-WES data 26
Supplementary Table 14 Enrichment tests of whether healthy *JAK2*-V617F carriers with low PGS (defined by median) were protected from MPN in the full UKBB-WES. 27
Supplementary Table 15 MPN predisposition risks of the germline variants associated with the five identified haematological traits, CH and MPN 28

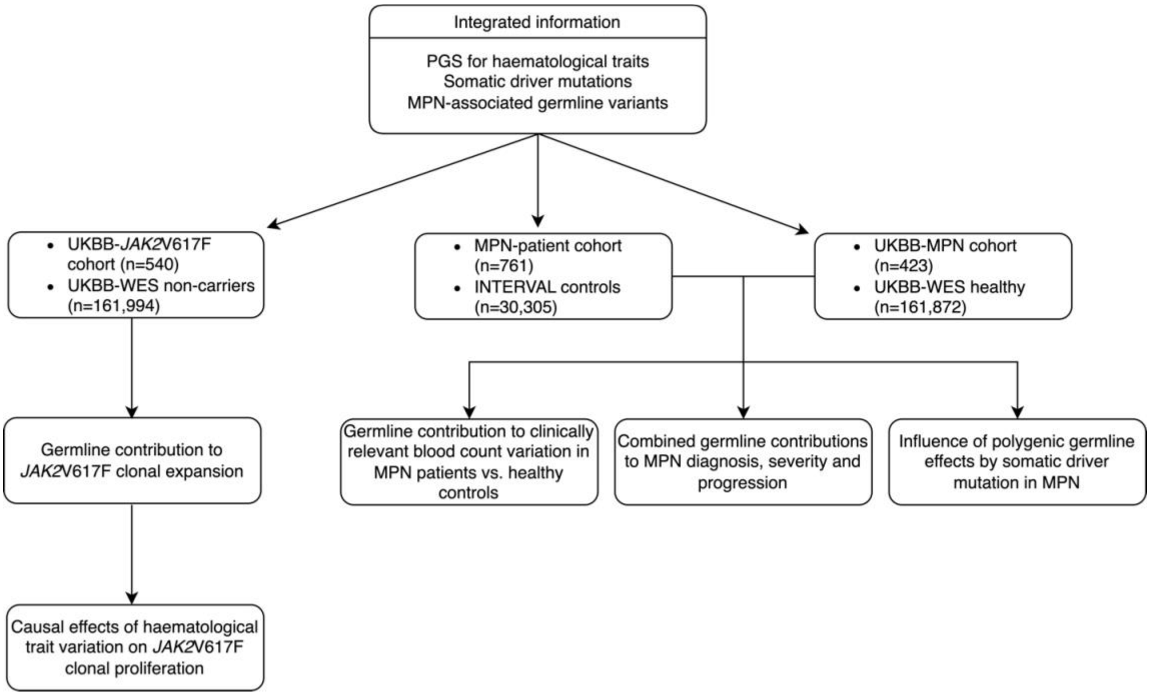

Supplementary Figure 1 Flowchart of study design and data sets used in the study.

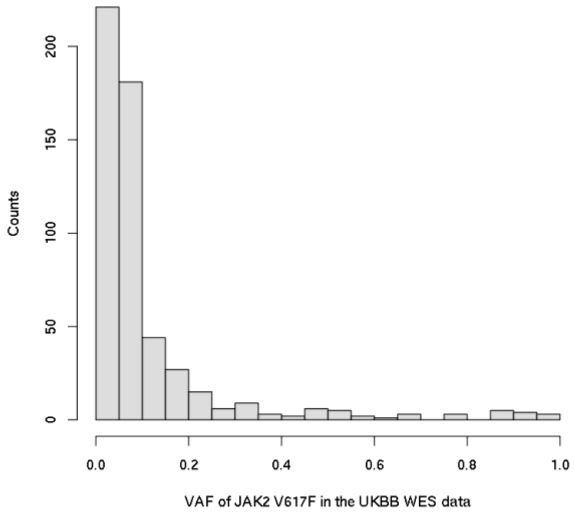

Supplementary Figure 2 Distribution of the VAF of *JAK2*-V617F G>T mutation in the genetically unrelated mutation carriers in the 200k UKBB-WES data (genetic relatedness <0.05; n = 540).

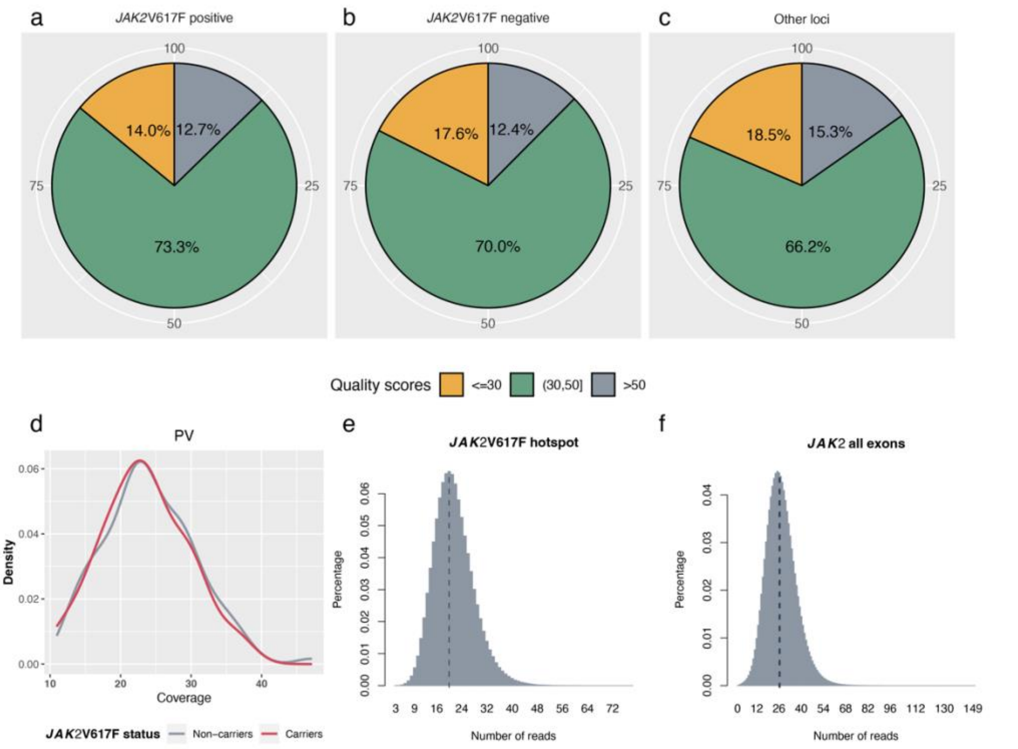

Supplementary Figure 3 Summary plot for *JAK2*-V617F mutation detection in the 200k UK Biobank WES data. Shown in order are the percentages of read quality scores in *JAK2*-V617F-positive (a), *JAK2*-V617F-negative (b) and other reads (c) in the same exon in *JAK2*. Coverage distribution within PV between *JAK2*-V617F carriers and non-carriers is shown in (d) and the distribution of coverage at the -V617F hotspot (mean=21.5, median=21, standard deviation [s.d.]=6.4) and all exons of *JAK2* (mean=27.5, median=27, s.d.=9.5) are shown in (e) and (f) respectively, with median values indicated by dash lines. The analysis of the pileup output (including read quality for low-quality reads) showed that the distribution of quality scores in the 200k UKBB samples was broadly comparable between *JAK2*-V617F positive, *JAK2*-V617F negative and other *JAK2* loci (a, b and c). We did not see a significant difference ($P_{\text{Difference}}$ = 0.53) in the coverage between PV-*JAK2*-V617F carriers and non-carriers (d).

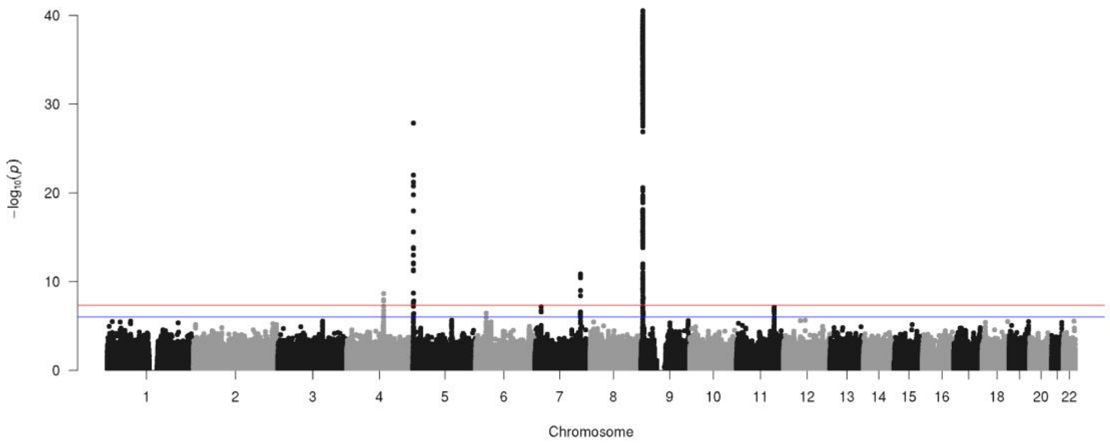

Supplementary Figure 4 Case-control GWAS of *JAK2*-V617F positivity in unrelated individuals in the UKBB-WES cohort (n = 1,125 cases vs. 338,919 controls). The genetics relatedness matrix was built on the after-imputation QC-ed variants after LD pruning with r^2 threshold of 0.2. Age, sex, WES batch and 10 PCs were fitted as covariates. The blue and red line represents the significance threshold of 10^{-6} and 5×10^{-8}.

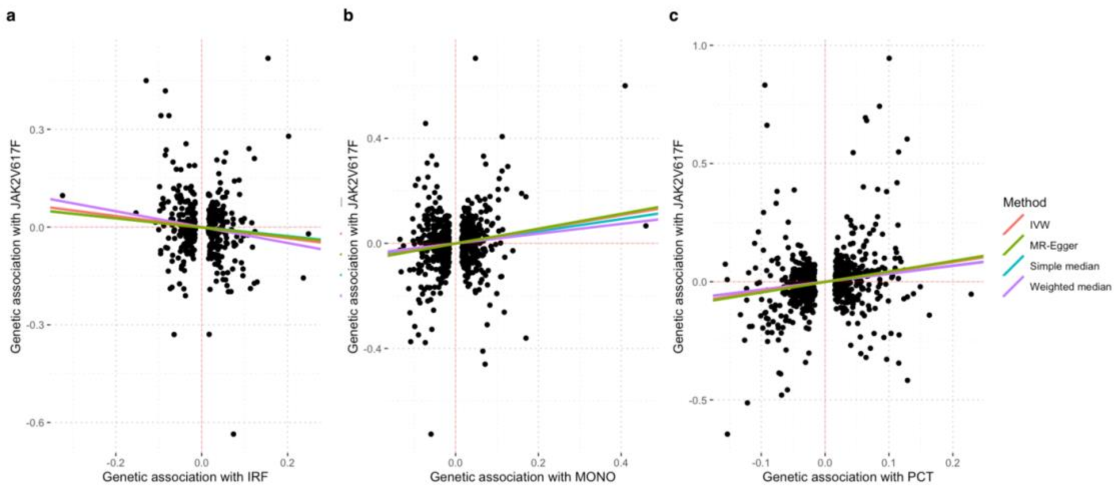

Supplementary Figure 5 MR plots for the forward causality between a blood cell trait and *JAK2*-V617F positivity. IVs for an exposure (e.g., IRF) were selected from the published GWAS in full UKBB with effect sizes (and standard errors [s.e.]) estimated in INTERVAL (association *P* <5e-8 in UKBB and also <0.05 in INTERVAL). The effect sizes for outcome (*JAK2*-V617F positivity) were based on the corresponding GWAS in full UKBB (n = 1,125 cases vs. 338,919 controls). The strongest dose-response relationship is for PCT, then for MONO and IRF, with the estimated Egger regression coefficients of 0.45 (s.e. = 0.17; *P* = 0.0075), 0.28 (s.e. = 0.13; *P* = 0.026) and -0.14 (s.e. = 0.16; *P* = 0.36) respectively. The *P*-values associated with the estimate coefficients were calculated based on Wald chi-squared tests. Lines shown were generated by the R function mr_plot() based on the causal effects estimated by four main MR methods indicated by four different colours.

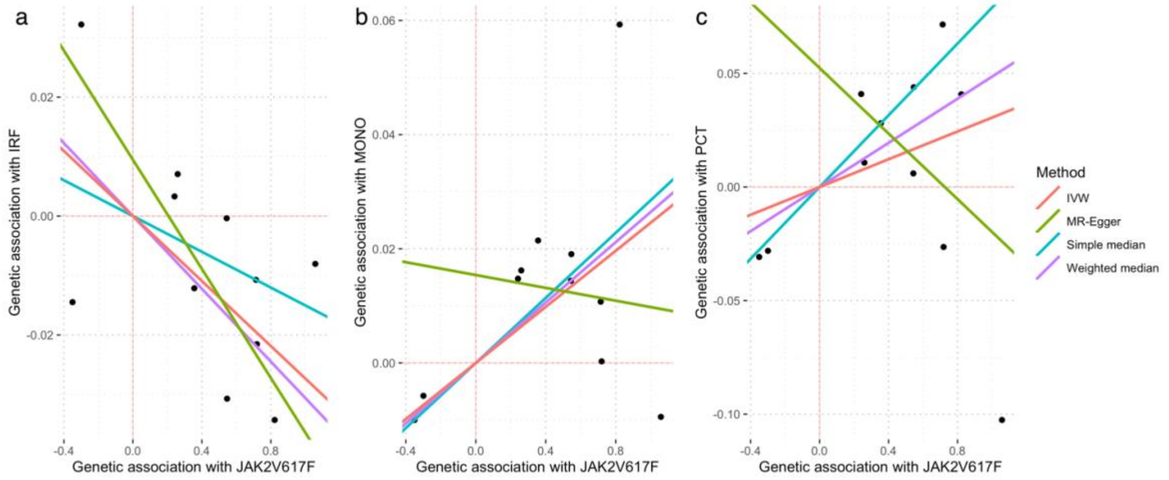

Supplementary Figure 6 MR plots for the reverse causality between *JAK2*-V617F positivity and a blood cell trait based on the independent IVs (LD r^2<0.05) selected from the GWAS for *JAK2*-V617F positivity in full UKBB (n = 1,125 cases vs. 338,919 controls) under association *P* < 1e-6. Lines shown were generated by the R function mr_plot() based on the causal effects estimated by four main MR methods indicated by four different colours.

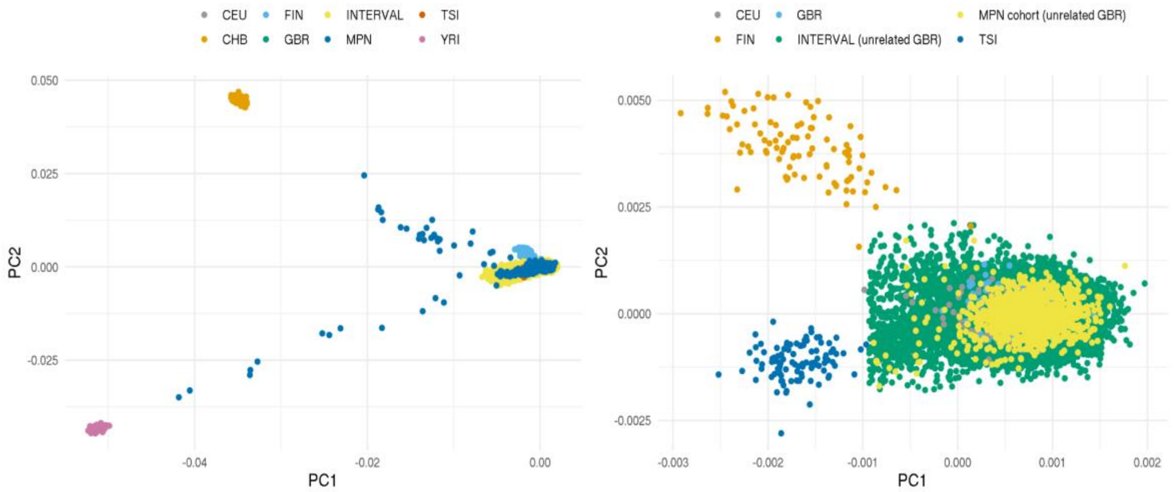

Supplementary Figure 7 Ancestry of individuals in the MPN-patient cohort and the control set INTERVAL. Shown are PC plots before (left panel) and after (right panel) removing British-ancestry (GBR) outliers and genetically related individuals (relatedness >0.05) in the MPN-patient cohort and the control set INTERVAL. We defined GBR-ancestry outliers as those who were more than 5 s.d. away from the mean of the GBR-1000 Genomes Project (GBR-1000G). The two datasets are shown in the background of global major populations in 1000G (left) and EUR-1000G (right) respectively. CEU, CEPH; FIN, Finnish; TSI, Toscani; CHB, Han Chinese; GBR, British; YRI, Yoruba

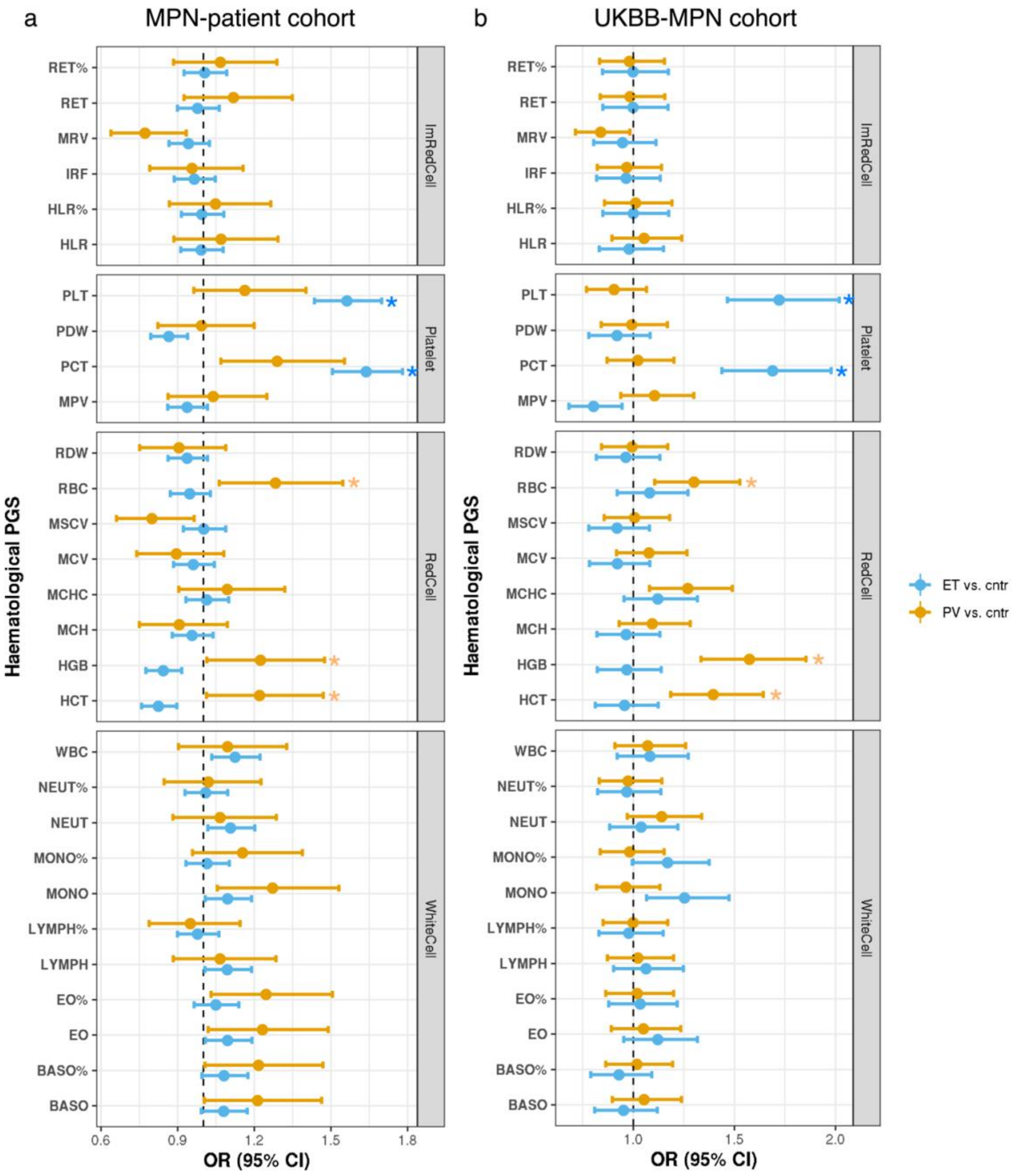

Supplementary Figure 8 Polygenic germline determinants of normal blood cell traits and their contribution to MPN classification (ET versus PV) in the MPN-patient cohort (a; n = 581 ET, 112 PV and 30,305 controls) and the UKBB-MPN cohort (b; n = 156 ET, 161 PV and 161,872 controls). Data are presented as ORs (changes in odds per s.d. increase of PGS; solid dots) with 95% CI (error bars). *indicate the significant haematological PGSs that were replicated in both datasets. Individual estimates (95% CI) in a) and b) are shown in Supplementary Table 10 and 11 respectively. Age, sex and 10 principal components were fit as covariates for both analyses. VAF of *JAK2*-V617F and sample batch were two additional covariates in the UKBB-MPN analysis. cntr, controls. Blood cell trait abbreviations detailed in Supplementary Table 2.

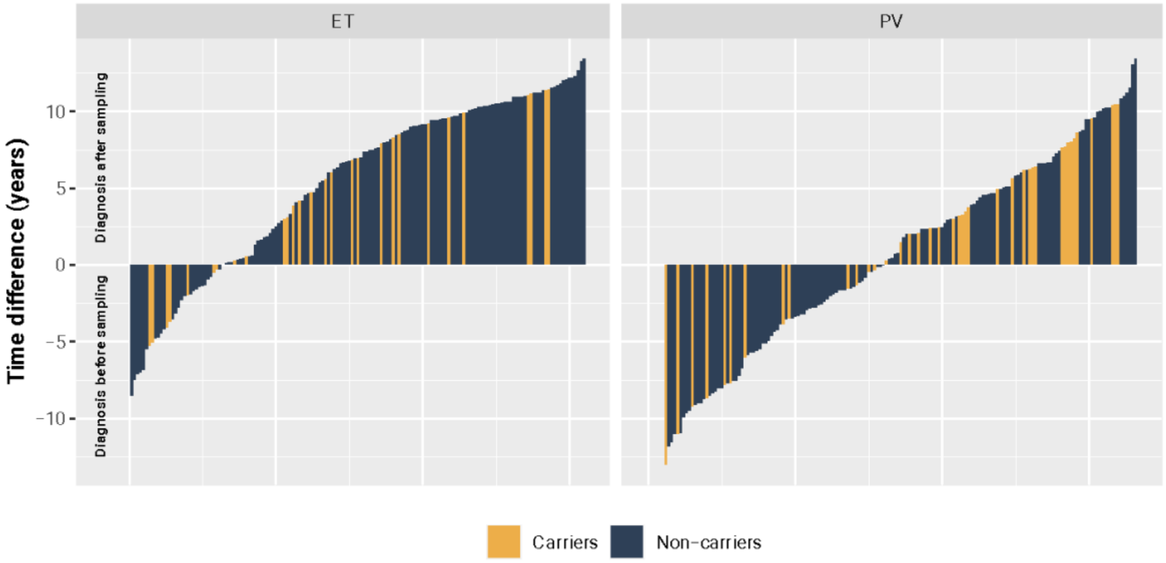

Supplementary Figure 9 Time difference from blood sample collected to diagnosis in years for ET (n = 155) and PV (n = 161) patients in UKBB-MPN cohort. Note that the sample size of ET is one less than that in the main analysis (n = 156) because of the unavailability of the time of blood sample collected. There were 28 *JAK2*-V617F carriers (21 with diagnosis after sampling) across 155 individuals with ET and 40 *JAK2*-V617F carriers (27 with diagnosis after sampling) across 161 individuals with PV (highlighted in yellow).

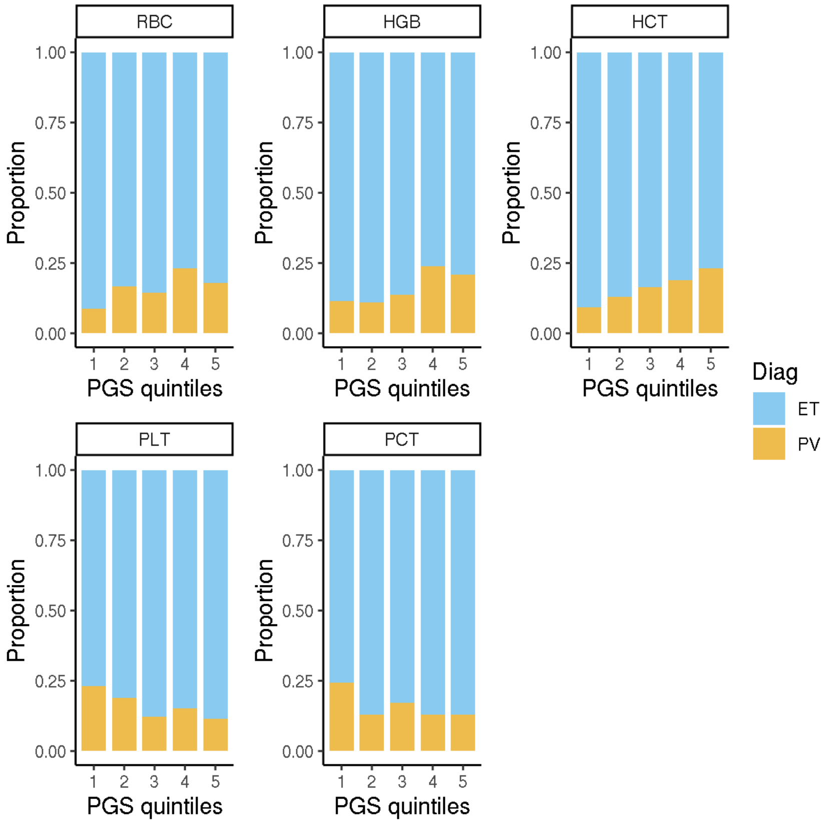

Supplementary Figure 10 Proportions of PV and ET patients in the MPN-patient cohort (n = 581 for ET and 112 for PV) in each PGS quintile across five significant blood cell traits associated with MPN classification at diagnosis.

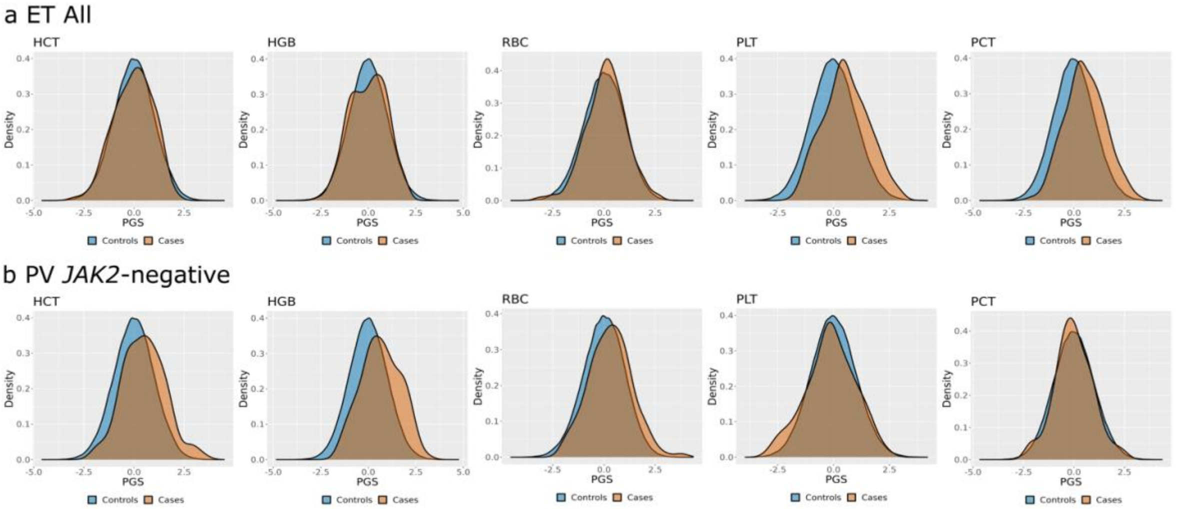

Supplementary Figure 11 PGS distributions for the five identified haematological traits between cases in the UKBB-MPN cohort and controls in UKBB (n = 158 ET and 121 PV *JAK2*-negative vs. 161,872 controls).

Supplementary Table 1 Summary table of the UKBB-WES data across *JAK2*-V617F mutant reads groups.

Features	#reads=0	#reads=1	#reads>=2	Total
All	161994	359	181	162534
Age>60	74452	182	125	74759
Male	77088	195	107	77390
ET	128	17	11	156
PV	121	11	29	161
MF	102	0	4	106

Supplementary Table 2 Full names and abbreviations of 29 blood cell traits involved in the study.

Abbreviation	Full name
PLT	Platelet count
MPV	Mean platelet volume
PDW	Platelet distribution width
PCT	Plateletcrit
RBC	Red blood cell count
MCV	Mean corpuscular volume
HCT	Haematocrit
MCH	Mean corpuscular haemoglobin
MSCV	Mean sphered corpuscular volume
MCHC	Mean corpuscular haemoglobin concentration
HGB	Haemoglobin concentration
RDW	Red cell distribution width
RET	Reticulocyte count
RET%	Reticulocyte fraction of red cells
IRF	Immature fraction of reticulocytes
HLR	High light scatter reticulocyte count
HLR%	High light scatter reticulocyte percentage of red cells
MRV	Mean reticulocyte volume
MONO	Monocyte count
NEUT	Neutrophil count
EO	Eosinophil count
BASO	Basophil count
LYMPH	Lymphocyte count
WBC	White blood cell count
MONO%	Monocyte percentage of white cells
NEUT%	Neutrophil percentage of white cells
EO%	Eosinophil percentage of white cells
BASO%	Basophil percentage of white cells
LYMPH%	Lymphocyte percentage of white cells

Measurements and descriptions are detailed in Vuckovic *et al.*

Supplementary Table 3 Germline polygenic associations with *JAK2*-V617F positivity in the UKBB-*JAK2*-V617F cohort (corresponding to Figure 1a). *P*-values were obtained from multinomial logistic regression analysis.

PGS	Small clones (VAF < 0.1)				Large clones (VAF > 0.1)			
	OR	95% CI		*P*	OR	95% CI		*P*
**MRV**	0.84	0.76	0.93	6.2E-04	0.85	0.71	1.01	0.062
**IRF**	0.85	0.77	0.94	0.0018	0.96	0.81	1.14	0.640
**MONO**	1.16	1.05	1.29	0.0036	1.20	1.02	1.42	0.033
**PCT**	1.19	1.07	1.32	9.5E-04	1.31	1.10	1.55	0.0022
**CH**	1.09	0.99	1.21	0.089	1.29	1.09	1.53	0.0028
**MPN-46/1**	1.35	1.23	1.48	1.5E-10	1.96	1.69	2.28	1.5E-18
**MPN-other**	1.46	1.33	1.61	3.8E-15	2.01	1.73	2.32	2.1E-20

Supplementary Table 4 Germline polygenic associations with *JAK2*-V617F positivity in the UKBB-*JAK2*-V617F cohort excluding MPN cases (n cases = 349 and 49 for small and large clones respectively). *P*-values were obtained from multinomial logistic regression analysis.

PGS	Small clones (VAF < 0.1)				Large clones (VAF > 0.1)			
	OR	95% CI		*P*	OR	95% CI		*P*
**MRV**	0.88	0.79	0.98	0.020	0.79	0.59	1.07	0.13
**IRF**	0.88	0.79	0.98	0.023	0.79	0.58	1.07	0.13
**MONO**	1.16	1.04	1.29	0.0080	1.16	0.86	1.56	0.32
**PCT**	1.16	1.04	1.30	0.0074	1.06	0.78	1.43	0.73
**CH**	1.05	0.95	1.18	0.34	1.11	0.83	1.49	0.49
**MPN-46/1**	1.31	1.18	1.44	1.7E-07	1.45	1.11	1.90	0.0069
**MPN-other**	1.38	1.25	1.53	6.6E-10	2.11	1.65	2.71	2.9E-09

Supplementary Table 5 Germline polygenic associations with *JAK2*-V617F positivity in the *JAK2*-V617F carriers in the full set of UKBB-WES. *P*-values were obtained from multinomial logistic regression analysis.

PGS	Small clones (VAF < 0.1)				Large clones (VAF > 0.1)			
	OR	95% CI		*P*	OR	95% CI		*P*
**MRV**	0.93	0.87	1.00	0.061	0.87	0.77	0.97	0.013
**IRF**	0.93	0.87	1.00	0.052	0.94	0.84	1.06	0.32
**MONO**	1.09	1.02	1.18	0.013	1.20	1.07	1.34	0.0014
**PCT**	1.15	1.07	1.24	1.4E-04	1.13	1.01	1.26	0.035
**CH**	1.09	1.01	1.17	0.019	1.22	1.10	1.37	3.1E-04
**MPN-46/1**	1.33	1.24	1.42	2.3E-17	1.92	1.74	2.12	9.7E-39
**MPN-other**	1.53	1.43	1.64	1.4E-36	2.01	1.83	2.21	5.9E-47

Supplementary Table 6 Forward causal inference of *JAK2*-V617F positivity across four main MR methods based on the *JAK2*-V617F GWAS with 540 cases vs. 161,994 controls in UKBB-WES. *P*-values were generated by R functions for each specific MR method (e.g., mr_ivw) based on Wald chi-squared tests.

Outcome	Exposure	#Instruments	Methods	OR (95% CI)	*P*
***JAK2*-V617F**	IRF	373	Simple median	0.76 (0.53-1.07)	0.12
			Weighted median	0.8 (0.55-1.17)	0.25
			IVW	0.78 (0.62-0.98)	0.032
			Egger	0.78 (0.52-1.18)	0.247
	MONO	688	Simple median	1.17 (0.88-1.56)	0.282
			Weighted median	1.2 (0.89-1.61)	0.231
			IVW	1.33 (1.11-1.6)	0.002
			Egger	1.47 (1.04-2.09)	0.031
	PCT	703	Simple median	1.51 (1.15-1.98)	0.003
			Weighted median	1.22 (0.91-1.63)	0.183
			IVW	1.51 (1.24-1.83)	4.1E-5
			Egger	1.17 (0.79-1.75)	0.435

Supplementary Table 7 Forward causal inference of *JAK2*-V617F positivity across four main methods based on the *JAK2*-V617F GWAS in full UKBB (n = 1,125 cases vs. 338,919 controls). *P*-values were generated by R functions for each specific MR method (e.g., mr_ivw) based on Wald chi-squared tests. The significant causal estimates for PCT and MONO were not biased by any potential pleiotropic outlier variants and were highly consistent with outlier-corrected causal estimates ($OR_{Outlier\text{-}corrected}$=1.46, 95% CI=1.27-1.67, $P=7.2\times10^{-8}$ for PCT; $OR_{Outlier\text{-}corrected}$=1.30, 95% CI=1.14-1.48, $P=6.2\times10^{-5}$ for MONO).

Outcome	Exposure	#Instruments	Methods	OR (95% CI)	*P*
JAK2-V617F	IRF	373	Simple_median	0.87 (0.69-1.11)	0.26
			Weighted_median	0.78 (0.61-1.01)	0.058
			IVW	0.84 (0.71-1)	0.047
			Egger	0.87 (0.64-1.18)	0.36
	MONO	688	Simple_median	1.26 (1.03-1.54)	0.024
			Weighted_median	1.2 (0.96-1.51)	0.10
			IVW	1.31 (1.15-1.49)	4.6E-05
			Egger	1.33 (1.03-1.71)	0.026
	PCT	703	Simple_median	1.41 (1.16-1.71)	6.1E-04
			Weighted_median	1.4 (1.13-1.72)	1.7E-03
			IVW	1.52 (1.29-1.78)	3.0E-07
			Egger	1.56 (1.13-2.17)	7.5E-03

Supplementary Table 8 Forward causal inference of *JAK2*-V617F positivity across four main methods with *JAK2*-V617F GWAS in full UKBB (n = 1,125 vs. 338,919) excluding instruments that are either associated with *JAK2*-V617F positivity at P <1e-6, in LD (LD r2 >0.01) with or within +/-5Mb region of those variants. *P*-values were generated by R functions for each specific MR method (e.g., mr_ivw) based on Wald chi-squared tests.

**Outcome**	**Exposure**	**#Instruments**	**Methods**	**OR (95% CI)**	***P***
JAK2-V617F	IRF	319	Simple_median	0.9 (0.69-1.16)	0.40
			Weighted_median	0.76 (0.58-0.99)	0.042
			IVW	0.85 (0.71-1)	0.056
			Egger	0.81 (0.6-1.1)	0.17
	MONO	571	Simple_median	1.2 (0.96-1.49)	0.12
			Weighted_median	1.16 (0.91-1.46)	0.22
			IVW	1.28 (1.11-1.48)	7.19E-04
			Egger	1.31 (0.99-1.73)	0.061
	PCT	547	Simple_median	1.44 (1.15-1.81)	0.0014
			Weighted_median	1.53 (1.2-1.95)	5.24E-04
			IVW	1.61 (1.38-1.88)	1.10E-09
			Egger	1.97 (1.42-2.74)	4.59E-05

Supplementary Table 9 Reverse causal inference of *JAK2*-V617F positivity and haematological traits across four main methods (#instruments = 11 with association P <1e-6; LD r2 <0.05) based on the *JAK2*-V617F-positivity GWAS in full UKBB (n = 1,125 cases vs. 338,919 controls). *P*-values were generated by R functions for each specific MR method (e.g., mr_ivw) based on Wald chi-squared tests.

**Exposure**	**Outcome**	**Methods**	**beta (s.e.)**	***P***
JAK2-V617F	IRF	Simple median	-0.015 (0.012)	0.23
		Weighted median	-0.031 (0.01)	0.0019
		IVW	-0.027 (0.011)	0.010
		Egger	-0.046 (0.026)	0.082
		(intercept)	0.009 (0.012)	0.44
	MONO	Simple median	0.029 (0.012)	0.014
		Weighted median	0.026 (0.01)	0.010
		IVW	0.025 (0.008)	0.0018
		Egger	-0.006 (0.017)	0.74
		(intercept)	0.015 (0.008)	0.049
	PCT	Simple median	0.079 (0.014)	5.14E-08
		Weighted median	0.049 (0.015)	0.0011
		IVW	0.031 (0.022)	0.16
		Egger	-0.072 (0.041)	0.075
		(intercept)	0.052 (0.019)	0.0054

Supplementary Table 10 Regression coefficients of significant (P < 0.0011 after Bonferroni correction) demographic, somatic mutation or germline variables for haematological traits in the MPN-patient cohort (corresponding to Figure 2c). *P*-values were calculated using the t-statistic from the T distribution.

Trait	Variables	coef (s.e.)	*P*-value
HGB	PGS	0.14 (0.035)	6.0E-05
	Sex:F	-0.33 (0.07)	4.1E-06
	JAK2	0.5 (0.08)	7.0E-10
	Chr9	0.4 (0.11)	3.2E-04
HCT	PGS	0.18 (0.05)	1.4E-04
	Sex:F	-0.5 (0.09)	1.2E-07
	JAK2	0.75 (0.1)	2.6E-14
PLT	*CALR*	0.52 (0.09)	3.4E-09
	Chr9	-0.36 (0.11)	7.7E-04
WBC	PGS	0.17 (0.03)	2.1E-06
	Chr9	0.44 (0.11)	4.2E-05

Supplementary Table 11 Regression coefficients of *JAK2* and PGS (P < 0.0063 after Bonferroni correction) in the UKBB-MPN cohort (corresponding to Figure 2e). *P*-values were calculated using the t-statistic from the T distribution.

Trait	Variables	coef (s.e.)	*P*-value
HGB	*JAK2*	0.7 (0.23)	2.83E-03
	PGS	0.47 (0.08)	9.49E-09
HCT	*JAK2*	0.82 (0.23)	5.22E-04
	PGS	0.43 (0.08)	3.00E-08
PLT	*JAK2*	1.6 (0.25)	4.70E-10
	PGS	0.5 (0.08)	1.28E-09
WBC	*JAK2*	0.77 (0.18)	3.22E-05
	PGS	0.42 (0.06)	1.48E-11

Supplementary Table 12 Polygenic germline effects of normal blood cell traits on MPN in our MPN-patient cohort corresponding to Supplementary Figure 8a. *P*-values were obtained from multinomial logistic regression analysis.

Cell type	Abbreviation	ET				PV			
		OR	95% CI		*P*	OR	95% CI		*P*
Immature Red Cell	HLR	0.99	0.91	1.08	0.84	1.07	0.89	1.29	0.49
	HLR%	0.99	0.92	1.08	0.90	1.05	0.87	1.27	0.63
	IRF	0.96	0.89	1.05	0.39	0.96	0.79	1.16	0.64
	**MRV**	0.94	0.87	1.02	0.16	0.77	0.64	0.93	**0.010**
	RET	0.98	0.90	1.06	0.60	1.12	0.93	1.35	0.25
	RET%	1.01	0.92	1.09	0.90	1.07	0.88	1.29	0.50
Platelet	MPV	0.94	0.86	1.02	0.12	1.04	0.86	1.25	0.69
	**PCT**	1.64	1.51	1.78	**7.11E-31**	1.29	1.07	1.55	**0.010**
	**PDW**	0.86	0.80	0.94	**6.23E-04**	0.99	0.82	1.20	0.94
	**PLT**	1.56	1.44	1.70	**2.68E-25**	1.16	0.96	1.40	0.12
Mature Red Cell	**HCT**	0.82	0.76	0.90	**6.05E-06**	1.22	1.01	1.47	**0.040**
	**HGB**	0.84	0.78	0.92	**6.42E-05**	1.22	1.02	1.48	**0.030**
	MCH	0.96	0.88	1.04	0.29	0.91	0.75	1.10	0.31
	**MCHC**	1.01	0.93	1.10	0.76	1.09	0.91	1.32	0.35
	MCV	0.96	0.88	1.04	0.34	0.89	0.74	1.08	0.25
	**MSCV**	1.00	0.92	1.09	0.97	0.80	0.66	0.96	**0.020**
	**RBC**	0.95	0.87	1.03	0.20	1.28	1.06	1.55	**0.010**
	RDW	0.94	0.86	1.02	0.12	0.90	0.75	1.09	0.29
White Cell	**BASO**	1.08	0.99	1.17	0.07	1.21	1.00	1.46	**0.050**
	**BASO%**	1.08	1.00	1.18	0.06	1.22	1.01	1.47	**0.040**
	**EO**	1.10	1.01	1.19	0.03	1.23	1.02	1.49	**0.030**
	**EO%**	1.05	0.97	1.14	0.26	1.25	1.03	1.51	**0.020**
	LYMPH	1.09	1.01	1.19	0.03	1.07	0.88	1.29	0.51
	LYMPH%	0.98	0.90	1.06	0.59	0.95	0.79	1.15	0.59
	**MONO**	1.09	1.01	1.19	0.03	1.27	1.05	1.53	**0.010**
	MONO%	1.01	0.93	1.10	0.74	1.15	0.96	1.39	0.13
	NEUT	1.11	1.02	1.20	0.02	1.07	0.88	1.29	0.51
	NEUT%	1.01	0.93	1.10	0.83	1.02	0.85	1.23	0.84
	WBC	1.12	1.03	1.22	**6.07E-03**	1.10	0.90	1.33	0.35

Values in bold indicate significant *P*-value at FDR-adjusted *P* < 0.05 for ET or *P* < 0.05 for PV.

Supplementary Table 13 Polygenic germline effects of normal blood cell traits on MPN in the UKBB-WES data corresponding to Supplementary Figure 8b. *P*-values were obtained from multinomial logistic regression analysis.

Cell type	Abbreviation	ET				PV			
		**OR**	**95% CI**		***P***	**OR**	**95% CI**		***P***
Immature Red Cell	HLR	0.98	0.83	1.15	0.80	1.05	0.90	1.24	0.53
	HLR%	1.00	0.85	1.18	0.99	1.01	0.86	1.19	0.88
	IRF	0.96	0.82	1.13	0.66	0.97	0.82	1.14	0.70
	MRV	0.95	0.81	1.11	0.51	0.84	0.71	0.99	0.03
	RET	1.00	0.85	1.17	0.99	0.98	0.84	1.16	0.85
	RET%	1.00	0.85	1.17	0.99	0.98	0.83	1.15	0.82
Platelet	MPV	0.80	0.68	0.95	0.01	1.11	0.94	1.30	0.23
	**PCT**	1.69	1.44	1.98	**1.14E-10**	1.02	0.87	1.20	0.78
	PDW	0.92	0.78	1.08	0.32	0.99	0.84	1.17	0.93
	**PLT**	1.72	1.47	2.02	**2.73E-11**	0.91	0.77	1.07	0.23
Mature Red Cell	**HCT**	0.96	0.81	1.12	0.59	1.40	1.19	1.64	**6.23E-05**
	**HGB**	0.97	0.82	1.14	0.70	1.57	1.34	1.86	**5.65E-08**
	MCH	0.96	0.82	1.13	0.66	1.09	0.93	1.28	0.27
	**MCHC**	1.12	0.96	1.32	0.16	1.27	1.08	1.49	**0.0036**
	MCV	0.92	0.78	1.08	0.32	1.08	0.92	1.27	0.36
	MSCV	0.92	0.78	1.08	0.31	1.01	0.86	1.18	0.94
	**RBC**	1.08	0.92	1.27	0.34	1.30	1.11	1.53	**0.0014**
	RDW	0.96	0.82	1.13	0.65	0.99	0.84	1.17	0.94
White Cell	BASO	0.95	0.81	1.12	0.56	1.05	0.90	1.24	0.52
	BASO%	0.93	0.79	1.09	0.38	1.02	0.87	1.20	0.83
	EO	1.12	0.95	1.32	0.17	1.05	0.89	1.24	0.55
	EO%	1.03	0.88	1.22	0.68	1.02	0.87	1.20	0.82
	LYMPH	1.06	0.90	1.25	0.46	1.02	0.87	1.20	0.77
	LYMPH%	0.98	0.83	1.15	0.78	1.00	0.85	1.17	1.00
	MONO	1.25	1.07	1.47	0.01	0.96	0.82	1.13	0.65
	MONO%	1.17	1.00	1.38	0.06	0.98	0.84	1.15	0.83
	NEUT	1.04	0.88	1.22	0.64	1.14	0.97	1.34	0.11
	NEUT%	0.97	0.83	1.14	0.70	0.98	0.83	1.14	0.76
	WBC	1.08	0.92	1.27	0.33	1.07	0.91	1.26	0.40

Values in bold indicate significant *P*-value at FDR-adjusted $P < 0.05$ for both ET and PV.

Supplementary Table 14 Enrichment tests of whether healthy *JAK2*-V617F carriers with low PGS (defined by median) were protected from MPN in the full UKBB-WES. *P*-values were calculated by Fisher's exact test.

**MPN**	**PGS**	**#Low PGS**		**#High PGS**		**OR (95% CI)**	***P***
		**Controls**	**Cases**	**Controls**	**Cases**		
ET	HCT	410	33	417	25	0.75 (0.42-1.32)	0.34
	HGB	408	35	419	23	0.64 (0.35-1.14)	0.13
	MONO	423	20	404	38	1.99 (1.11-3.67)	0.015
	**PCT***	427	16	400	42	2.80 (1.51-5.42)	3.8E-04
	**PLT***	425	18	402	40	2.35 (1.29-4.43)	0.0027
	RBC	417	26	410	32	1.25 (0.71-2.23)	0.42
PV	HCT	412	43	415	40	0.92 (0.57-1.49)	0.82
	HGB	413	42	414	41	0.97 (0.6-1.57)	1.00
	MONO	409	46	418	37	0.79 (0.49-1.27)	0.36
	PCT	416	39	411	44	1.14 (0.71-1.85)	0.65
	PLT	414	41	413	42	1.03 (0.64-1.66)	1.00
	RBC	413	42	414	41	0.97 (0.6-1.57)	1.00

*indicates the identified PGS with a significance *P*-value passed Bonferroni correction (=0.05/12).

Supplementary Table 15 MPN predisposition risks of the germline variants associated with the five identified haematological traits, CH and MPN (corresponding to Figure 3a).

PGS	MPN-patient cohort				UKBB-MPN cohort			
	ET		PV		ET		PV	
	**OR (95% CI)**	***P***	**OR (95% CI)**	***P***	**OR (95% CI)**	***P***	**OR (95% CI)**	***P***
**HCT**	0.81 (0.74-0.88)	6.25E-07	1.19 (0.99-1.44)	0.068	0.94 (0.80-1.11)	0.48	1.38 (1.17-1.62)	1.14E-04
**HGB**	0.83 (0.77-0.91)	2.44E-05	1.21 (1.00-1.47)	**0.047**	0.95 (0.81-1.12)	0.55	1.55 (1.32-1.83)	**1.52E-07**
**RBC**	0.89 (0.82-0.97)	0.0069	1.15 (0.96-1.39)	0.13	1.05 (0.89-1.23)	0.58	1.27 (1.08-1.49)	0.0034
**PLT**	1.53 (1.40-1.66)	**0**	1.13 (0.94-1.37)	0.20	1.66 (1.41-1.95)	**8.71E-10**	0.88 (0.75-1.04)	0.13
**PCT**	1.58 (1.45-1.72)	**0**	1.21 (1.00-1.47)	0.048	1.61 (1.37-1.89)	**9.55E-09**	0.99 (0.84-1.16)	0.87
**CH**	1.20 (1.08-1.32)	**5.11E-04**	1.16 (0.92-1.46)	0.22	1.19 (1.01-1.39)	**0.036**	1.11 (0.94-1.30)	0.21
**MPN-46/1**	1.28 (1.19-1.39)	3.98E-10	2.02 (1.71-2.40)	2.22E-16	0.98 (0.83-1.15)	0.76	1.14 (0.98-1.33)	0.097
**MPN-other**	1.32 (1.20-1.46)	**4.48E-08**	1.78 (1.42-2.23)	**5.43E-07**	1.43 (1.23-1.66)	**3.95E-06**	1.27 (1.09-1.48)	**0.0024**

The significant PGSs with *P* <0.05 in both cohorts are in bold.
