# Supplementary material for: Inherited polygenic effects on common hematological traits influence clonal selection on JAK2V617F and the development of myeloproliferative neoplasms
Source: Nat Genet. 2024 Jan 17;56(2):273–80. doi: 10.1038/s41588-023-01638-x (PMC10864174; doi:10.1038/s41588-023-01638-x)
Supplement: Supplementary file 2 — Reporting Summary [file 41588_2023_1638_MOESM2_ESM.pdf]

nature portfolio

Corresponding author(s): Dragana Vuckovic
Jyoti Nangalia
Nicole Soranzo

Last updated by author(s): Nov 7, 2023

Reporting Summary

Nature Portfolio wishes to improve the reproducibility of the work that we publish. This form provides structure for consistency and transparency in reporting. For further information on Nature Portfolio policies, see our Editorial Policies and the Editorial Policy Checklist.

Statistics

For all statistical analyses, confirm that the following items are present in the figure legend, table legend, main text, or Methods section.

n/a	Confirmed	
☐	☒	The exact sample size (*n*) for each experimental group/condition, given as a discrete number and unit of measurement
☐	☒	A statement on whether measurements were taken from distinct samples or whether the same sample was measured repeatedly
☐	☒	The statistical test(s) used AND whether they are one- or two-sided<br>*Only common tests should be described solely by name; describe more complex techniques in the Methods section.*
☐	☒	A description of all covariates tested
☐	☒	A description of any assumptions or corrections, such as tests of normality and adjustment for multiple comparisons
☐	☒	A full description of the statistical parameters including central tendency (e.g. means) or other basic estimates (e.g. regression coefficient) AND variation (e.g. standard deviation) or associated estimates of uncertainty (e.g. confidence intervals)
☐	☒	For null hypothesis testing, the test statistic (e.g. *F*, *t*, *r*) with confidence intervals, effect sizes, degrees of freedom and *P* value noted<br>*Give P values as exact values whenever suitable.*
☒	☐	For Bayesian analysis, information on the choice of priors and Markov chain Monte Carlo settings
☒	☐	For hierarchical and complex designs, identification of the appropriate level for tests and full reporting of outcomes
☐	☒	Estimates of effect sizes (e.g. Cohen's *d*, Pearson's *r*), indicating how they were calculated

Our web collection on statistics for biologists contains articles on many of the points above.

Software and code

Policy information about availability of computer code

Data collection	We did not collect individual-level data. All datasets used in the study were sourced from public available data (eg UKBB) or from data previously collected for published studies.<br><br>MPN patients data originated from Grinfeld et al. NEJM 2018 with somatic mutation data available for each individual. Clinical data were shared by the authors.<br>UKBB-WES data were obtained from UK Biobank repository. INTERVAL data were shared by authors from the INTERVAL cohort.
Data analysis	The case-control GWAS for JAK2V617F positivity in the UKBB-WES was conducted using SAIGE version 0.38 (https://github.com/weizhouUMICH/SAIGE). GRM computation for the case-control GWAS for JAK2V617F positivity and conditional analysis for the GWAS associations for 29 blood cell traits were carried out using GCTA-GRM (https://yanglab.westlake.edu.cn/software/gcta/#MakingaGRM) and GCTA-COJO (https://yanglab.westlake.edu.cn/software/gcta/#COJO) respectively in GCTA version 1.93.3beta2 (https://yanglab.westlake.edu.cn/software/gcta/#Download). PGS computation in MPN-patient cohort and INTERVAL, LD-based variant pruning and LD r2 computation were conducted in PLINK version 1.9 (https://www.cog-genomics.org/plink/). MR analysis for JAK2V617F positivity were performed using the R package MendelianRandomization version 0.5.1 (https://cran.r-project.org/src/contrib/Archive/MendelianRandomization/). Haematological traits variation explained by germline and somatic genetic features in MPN patients were estimated in R package MASS version 7.3-57 (https://cran.r-project.org/src/contrib/Archive/MASS/), car version 3.0-10 (https://cran.r-project.org/src/contrib/Archive/car/) and boot version 1.3-28 (https://cran.r-project.org/src/contrib/Archive/boot/).

For manuscripts utilizing custom algorithms or software that are central to the research but not yet described in published literature, software must be made available to editors and reviewers. We strongly encourage code deposition in a community repository (e.g. GitHub). See the Nature Portfolio guidelines for submitting code & software for further information.

Data

Policy information about availability of data

All manuscripts must include a data availability statement. This statement should provide the following information, where applicable:

- Accession codes, unique identifiers, or web links for publicly available datasets
- A description of any restrictions on data availability
- For clinical datasets or third party data, please ensure that the statement adheres to our policy

GWAS summary statistics for the 29 blood cell traits were sourced from a published study (Vuckovic et al. Cell [2020]) and are available at GWAS Catalog (https://www.ebi.ac.uk/gwas/) with accession numbers GCST90002379–GCST90002407. Individual-level phenotype/genotype data of UK Biobank can be requested via application to https://www.ukbiobank.ac.uk. Individual-level genotype data of the 1000 Genomes Project are available at https://www.internationalgenome.org/. INTERVAL data can be requested via application to the study leaders (https://www.intervalstudy.org.uk/) and MPN-patient cohort data can be requested from the authors.

Human research participants

Policy information about studies involving human research participants and Sex and Gender in Research.

Reporting on sex and gender	Only sex was considered in the study with sex information determined based on individual-level genotype data using the sex check tool implemented in PLINK v1.9 (https://www.cog-genomics.org/plink/).<br>No sex-specific analysis were performed but sex as a covariate was always fitted in the model for identifying any potential sex effect.
Population characteristics	Age and sex:<br>In the 200k UKBB-WES data (n=162,534), 46% individuals are aged at greater than 60 years (n=74,759) and 48% are males (n=74,759).<br>In the MPN-patient cohort (n=761), 45% are aged at >60 years (n=342) and 45% are males (n=339).<br>In the healthy cohort INTERVAL (n=30,305), the median age is 43.3 with only 13% are aged at >60 years (n=3965); 50% are males (n=15,089).<br><br>Diagnosis:<br>In the 200k UKBB-WES data (n=162,534), 46% individuals are aged at greater than 60 years (n=74,759), 48% are males (n=74,759) and 423 individuals have a diagnosis of MPN including 156 ET, 161 PV and 106 MF. We identified known MPN cases through annotations for essential thrombocythemia (ET; ICD10 D47.3, D75.2), polycythaemia (PV; ICD10 D45), myelofibrosis (MF; ICD10 D47.4, D75.81), chronic myeloid leukaemia (CML; ICD10 C921, C922, C931), and chronic myeloproliferative disease (CMD; ICD10 D47.1).<br>In the MPN-patient cohort (n=761), 45% are aged at >60 years (n=342), 45% are males (n=339). All the individuals in this cohort are MPN patients including 581 ET, 112 PV and 68 MF.<br>For both the MPN-patient cohort and the MPN cases in UKBB-WES (“UKBB-MPN cohort”), we only included MPN patients who were explicitly diagnosed as ET, PV or MF; patients with conflicting records/unclassified/other MPN (e.g., chronic myeloid leukaemia), were excluded (n=761 and 423 respectively with genotype data available).<br><br>Time of the diagnosis and blood sampling of UKBB - MPN cohort:<br>The MPN cases in the UKBB-MPN cohort were not restricted to participants who had existing diagnoses at the time of blood sampling. Any participant whose inpatient records could be matched to the MPN ICD10 codes were included as cases in this study. To calculate the time interval from blood draw to MPN diagnosis, we matched MPN cases to the information regarding the date a blood sample collected and when a particular diagnosis was first recorded in the hospital data (episode start date) downloaded from the UKBB Data Portal. If an MPN case could be matched to multiple episode start dates, the earliest date was selected as the diagnosis date. We then calculated the time interval between the two dates for ET and PV respectively.<br><br>Genetic relatedness and ancestry:<br>Throughout the study, we only include genetically unrelated individuals and those that are of European ancestry. Please see below Data Exclusions for detailed inclusion cut-off in the raw data processing within this study.
Recruitment	UK Biobank analyses were undertaken under application numbers 56844 and 13745. MPN patient samples were obtained following written informed consent and ethics approval as described previously22. Briefly, patient samples with MPN were collected from outpatient clinics at Addenbrooke’s Hospital, Guys and St Thomas’ Hospital in the UK, under the clauses of the ‘Causes of Clonal Haematological Disorders Project’ which had regional ethical approval from the Eastern Multi-region Ethics Committee (MREC 02/5/22 and 07/MRE05/44) and local research and ethical approval at participating UK hospitals. Additional MPN samples were obtained and the University of Florence Careggi Hospital, Italy with local ethics approval. Whole blood derived samples were additionally analysed from the Primary Thrombocythaemia -1 (PT1) trials. PT1 is a multi-center international trial in ET. Analyses for this study was conducted under the Cambridge Blood and Stem Cell Biobank ethics, 18/EE/0199 expiry 14/07/2024.
Ethics oversight	Ethics oversight was provided by the Cambridge Stem Cell Biobank and also ethical board review at Wellcome Sanger Institute prior to sample sequencing.

Note that full information on the approval of the study protocol must also be provided in the manuscript.

Field-specific reporting

Please select the one below that is the best fit for your research. If you are not sure, read the appropriate sections before making your selection.

☒ Life sciences ☐ Behavioural & social sciences ☐ Ecological, evolutionary & environmental sciences

For a reference copy of the document with all sections, see nature.com/documents/nr-reporting-summary-flat.pdf

Life sciences study design

All studies must disclose on these points even when the disclosure is negative.

Sample size

No sample-size calculation was performed.

In the discovery analysis for the JAK2-V617F positivity, we used the 200k UKBB-WES data sourced from a published study Kar et al. Nat Genet 2022 which included all the UKBB participants for whom whole exome sequencing data have been released in December 2020.

The disease classification analysis were based on the discovery dataset we called "MPN-patient cohort". We included all the MPN-patient data sourced from a published study Grinfeld et al, NEJM 2018 only excluding those that did not pass genotyping/imputation QC, genetic relatedness and ancestry (see below). We then replicate the findings in the 200k UKBB-WES data obtained from the UK Biobank repository.

Below are the exact sample sizes in each dataset:
UKBB-JAK2-V617F cohort (n=540) with non-carriers in the 200k UKBB-WES (n=161,994)
JAK2-V617F positivity in full UKBB-WES (n=1,125 cases; 338,919 controls)
MPN-patient cohort (n=761) with healthy controls in INTERVAL (n=30,305)
UKBB-MPN cohort (n=423) with healthy controls in the 200k UKBB-WES (n=161,872)

Data exclusions

For both the MPN-patient cohort and the MPN cases in UKBB-WES (“UKBB-MPN cohort”), we only included MPN patients who were explicitly diagnosed as ET, PV or MF; patients with conflicting records, unclassified or other MPN, such as chronic myeloid leukaemia, were excluded (n = 761 and 423 respectively with genotype data available). UKBB-WES data was also used to identify a set of individuals (n = 540, of which 72 had a corresponding diagnosis of ET, PV or MF, 6 CMD, 3 CML, 63 with more than one records of MPN subtypes, and 396 healthy) with #mutant reads (either at least 1 or =>2; n = 359 and 181 respectively) corresponding to the JAK2V617F mutation (“UKBB-JAK2V617F cohort”) – these patients comprised both those with and without a formal diagnosis of MPN in UKBB. The mpileup function of Samtools 1.9 was used with the FASTA file of GRCh38 assembly and the parameter "-r chr9:5073767-5073775" to calculate the number of mutant reads and coverage of each base around the V617 hotspot. We excluded reads with base quality <13 using the base quality filter in Samtools mpileup tool. JAK2V617F clone size was measured by VAF in the UKBB-WES cohort. Genotype data inclusion criteria are described above.

We excluded samples in the MPN-patient cohort (n=1,358) with an outlying heterozygosity rate >+/- 3 s.d. and high IBD sharing >0.9 and obtained 1,207 samples. In the combined dataset of MPN cases, INTERVAL and 1000 Genomes Project (1000G) with global major populations, we removed individuals >5 s.d. from the mean of the British ancestry (GBR-1000G) and obtained 1,010 MPN patients and 30,949 control individuals from INTERVAL.

Replication

All the Results were first described in MPN-patient cohort (with INTERVAL cohort as healthy controls) and then replicated in the UKBB-MPN cohort (with UKBB healthy as controls).We can't replicate the germline association with JAK2V617F positivity in the UKBB-JAK2V617F cohort (with UKBB-WES non carriers as controls) in MPN-patient cohort because of the unavailability of the measurements on JAK2 mutations in the control dataset INTERVAL.

Randomization

No randomization was applied. For identifying any germline associations, we fit age, sex, PCs (to control for potential population stratification), sample batch and VAF of JAK2V617F as covariates wherever applicable.

Blinding

No blinding was undertaken.

Reporting for specific materials, systems and methods

We require information from authors about some types of materials, experimental systems and methods used in many studies. Here, indicate whether each material, system or method listed is relevant to your study. If you are not sure if a list item applies to your research, read the appropriate section before selecting a response.

Materials & experimental systems

n/a	Involved in the study
☒	☐ Antibodies
☒	☐ Eukaryotic cell lines
☒	☐ Palaeontology and archaeology
☒	☐ Animals and other organisms
☒	☐ Clinical data
☒	☐ Dual use research of concern

Methods

n/a	Involved in the study
☒	☐ ChIP-seq
☒	☐ Flow cytometry
☒	☐ MRI-based neuroimaging
